# Computational molecular docking and virtual screening revealed promising SARS-CoV-2 drugs

**DOI:** 10.1093/pcmedi/pbab001

**Published:** 2021-01-18

**Authors:** Maryam Hosseini, Wanqiu Chen, Daliao Xiao, Charles Wang

**Affiliations:** Center for Genomics, School of Medicine, Loma Linda University, Loma Linda, CA 92350, USA; Center for Genomics, School of Medicine, Loma Linda University, Loma Linda, CA 92350, USA; Lawrence D. Longo, MD Center for Perinatal Biology, Department of Basic Sciences, School of Medicine, Loma Linda University, Loma Linda, CA 92350, USA; Center for Genomics, School of Medicine, Loma Linda University, Loma Linda, CA 92350, USA; Division of Microbiology & Molecular Genetics, Department of Basic Sciences, School of Medicine, Loma Linda University, Loma Linda, CA 92350, USA

**Keywords:** COVID-19, SARS-CoV-2, Mpro, PLpro, RdRp, virtual screening, molecular docking

## Abstract

The pandemic of novel coronavirus disease 2019 (COVID-19) has rampaged the world, with more than 58.4 million confirmed cases and over 1.38 million deaths across the world by 23 November 2020. There is an urgent need to identify effective drugs and vaccines to fight against the virus. Severe acute respiratory syndrome coronavirus 2 (SARS-CoV-2) belongs to the family of coronaviruses consisting of four structural and 16 non-structural proteins (NSP). Three non-structural proteins, main protease (Mpro), papain-like protease (PLpro), and RNA-dependent RNA polymerase (RdRp), are believed to have a crucial role in replication of the virus. We applied computational ligand-receptor binding modeling and performed comprehensive virtual screening on FDA-approved drugs against these three SARS-CoV-2 proteins using AutoDock Vina, Glide, and rDock. Our computational studies identified six novel ligands as potential inhibitors against SARS-CoV-2, including antiemetics rolapitant and ondansetron for Mpro; labetalol and levomefolic acid for PLpro; and leucal and antifungal natamycin for RdRp. Molecular dynamics simulation confirmed the stability of the ligand-protein complexes. The results of our analysis with some other suggested drugs indicated that chloroquine and hydroxychloroquine had high binding energy (low inhibitory effect) with all three proteins—Mpro, PLpro, and RdRp. In summary, our computational molecular docking approach and virtual screening identified some promising candidate SARS-CoV-2 inhibitors that may be considered for further clinical studies.

## Introduction

A novel coronavirus disease (COVID-19) started in December 2019^1,2^ and spreaded to more than 230 countries across the world, with the ongoing pandemic becoming a global health emergency.^3^ As of 23 November 2020, there have been more than 58.4 million confirmed cases and 1.38 million deaths globally.^3^ Similar to the SARS (Severe Acute Respiratory Syndrome) coronavirus and MERS (Middle East Respiratory Syndrome) coronavirus, the SARS-CoV-2 (Severe Acute Respiratory Syndrome 2) belongs to the betacoronavirus (beta-CoV) family, RNA viruses with crown-like spikes on the surface of the coronavirus particles. The fatality rate of the new SARS-CoV-2 seems lower than that of SARS and MERS. An estimate of the overall fatality for SARS-CoV-2 is 2%–3%,^4^ whereas the World Health Organization (WHO) estimated a fatality rate of ∼14%–15% for SARS^5^ and ∼35% for MERS.^6^ However, SARS-CoV-2 has features of rapid transmission from person-to-person, asymptomatic transmission^7^ and prolonged symptomatic development, as well as substantially increased fatalities in the aged group.^8^ The pandemic of COVID-19 has caused a surge in the need for intensive care, which has caused tremendous pressure on the healthcare systems in many countries.

Substantial efforts have been made in treatment of patients infected with SARS-CoV-2.^9–11^ The anti-coronaviral strategies include preventing the synthesis of viral RNA, inhibiting virus replication, blocking the virus binding to human cell receptors, or inhibiting the virus's self-assembly process.^12^ The SARS-CoV-2 genome encodes four structural and 16 non-structural proteins (NSP).^13^ Among these translated NSPs, the main protease (Mpro, EC 3.4.22.69), also called chymotrypsin-like protease (3C-like protease), and the papain-like protease (PLpro, EC 3.4.19.12) are two essential proteases for proteolytic processing of the coronavirus replicase polyprotein therefore generating functional replication complex of the virus^14,15^; whereas RNA-dependent RNA polymerase (RdRp, EC 2.7.7.48) is the central enzyme for RNA synthesis in all positive-strand RNA virus replication.^16^ These three NSP proteins play crucial roles in coronavirus replication, making them attractive targets for anti-coronaviral drug design. Targeting one or multiple NSP proteins including Mpro, RdRp, and PLpro, may lead to potential treatment for COVID-19.

Dozens of potential therapies for SARS-CoV-2 have been suggested during the COVID-19 outbreaks. The WHO launched a trial, SOLIDARITY, to focus on testing the four most promising COVID-19 treatments—remdesivir; chloroquine and hydroxy-chloroquine; lopinavir plus ritonavir; and lopinavir plus ritonavir and interferon-beta. It is worth mentioning that the four therapies against SARS-CoV-2 are somewhat targeting one of the three NSPs proteins of coronavirus—Mpro, RdRp, and PLpro. Chloroquine/hydroxy-chloroquine and lopinavir/ritonavir were removed from the COVID-19 treatment protocols in June 2020, because of possible risks and uncertainty of their benefits, but are still being studied in clinical trials.^17,18^ Remdesivir (Veklury) was the first and only drug approved by the FDA, on 22 October 2020, to treat COVID-19.^19^ As an approved human immunodeficiency virus (HIV) reverse transcriptase inhibitor, remdesivir was shown to decrease the recovery time for patients hospitalized with COVID-19 by targeting the SARS-CoV-2 RdRp enzyme.^20^ Lopinavir and ritonavir, which act against the viral main protease (Mpro), have been shown to be effective in treating patients with SARS^21^ and MERS-CoV.^22,23^ A randomized clinical trial of lopinavir–ritonavir efficacy in patients with COVID-19 has been carried out in Wuhan, China.^24^ Chloroquine and hydroxychloroquine, used as antimalarial drugs, have been proposed as a potential treatment for COVID-19^25,26^ despite no conclusive evidence of their benefit and safety. Recently, one report suggested that chloroquine and hydroxychloroquine might have some inhibitory activity against coronavirus papain-like protease.^27^

Computational screening of the FDA-approved drugs with the potential of targeting SARS-CoV-2 is a cost-effective and less time-consuming strategy and can quickly identify promising ready-to-use candidates. Recently, molecular docking and virtual screening methods have been attempted to identify potential drugs for COVID-19 by protein-ligand binding energy prediction. Because of the limitation in crystal structures for SARS-CoV-2 proteins, many studies have used homology models based on the SARS-CoV-2 genome and SARS crystal structure.^28,29^ Previous studies have screened the small molecules that target SARS-CoV-2 Mpro, PLpro, or RdRp proteins.^27–31^ Some potential candidates for SARS-CoV-2 have been identified, many of which are anti-HIV or hepatitis C (HCV) drugs. In this study, we aimed to screen FDA-approved drugs that may have inhibitory activity against one or more of the three SARS-CoV-2 proteins Mpro, RdRp, and PLpro, and attempted to identify other drug candidates that may have higher inhibitory activity and lower binding energies with the three SARS-CoV-2 proteins than remdesivir. In this regard, we conducted molecular docking and virtual screening of 1615 FDA-approved drugs on the binding pocket of SARS-CoV-2 Mpro, PLpro, and RdRp proteins.

## Methods

### Preparation of protein and ligand structures

To achieve the mode of interaction of the FDA-approved drugs with the binding pocket of three different SARS-CoV-2 NSPs, we prepared three-dimensional (3D) structures of Mpro, PLpro, and RdRp proteins. We performed molecular docking analysis on the FDA-approved drugs with the binding pocket of three different SARS-CoV-2 NSPs to identify potential drugs for the treatment of COVID-19. The structures of SARS-CoV-2 Mpro (PDB ID: 6LU7, Chain A, 2.16 Å)^32^ in a complex with N3 inhibitor and RdRp (PDB ID: 7BV2, Chain A, 2.50Å) in a complex with remdesivir monophosphate (RMP) were retrieved from the protein data bank (PDB) website (www.rcsb.org).^33^ A high-quality model of SARS-CoV-2 PLpro built based on the SARS-CoV-2 genome and SARS-CoV PLpro (PDB ID: 3E9S, 2.6 Å)^34^ crystal structure with GMQE and QMEAN scores of 0.9 and –0.29, respectively, was downloaded to be used as a PLpro receptor.^35^ Inhibitors N3 in 6LU7, TTT (5-amino-2-methyl-N-[(1R)-1-naphthalen-1-ylethyl] benzamide) in 3E9S, and remdesivir monophosphate (GS-441 524-MP) in 7BV2 were used as controls for Mpro, PLpro, and RdRp, respectively.

### Preparation of ligand structures

Three-dimensional structures of the FDA-approved drugs, containing 1615 compounds, were downloaded from the ZINC15 database^36^ in structure-data file (SDF) format. Protein structures were downloaded in PDB format.

For Glide, the ligand structures were prepared using LigPrep^37^ with default values at pH 7.2 ± 1.0. The protein structures were prepared using the Protein Preparation Wizard from Maestro Task with default settings. The ligand and protein structures to be used by rDock were generated using the same methods.

In the case of AutoDock Vina, three receptor molecules and 1615 ligands were prepared using AutoDockTools (ADT, v1.5.6)^38^ to be used as input files for the docking analysis. For the preparation of protein input files, all water molecules, ligands, and ions were removed, and polar hydrogens were added from the PDB file using the prepare_receptor4.py command of the ADT. Kollman-united charge was used to calculate the partial atomic charge and the prepared file was saved in a format to be used in the following steps. Then, OpenBabel (v2.3.1)^39^ was used to separate the files, add hydrogen bonds and assign rotatable bonds and Gasteiger-Marsili charges. Finally, all the ligands were saved in PDBQT for further docking processes.

### Molecular docking and virtual screening

**Figure 1.**
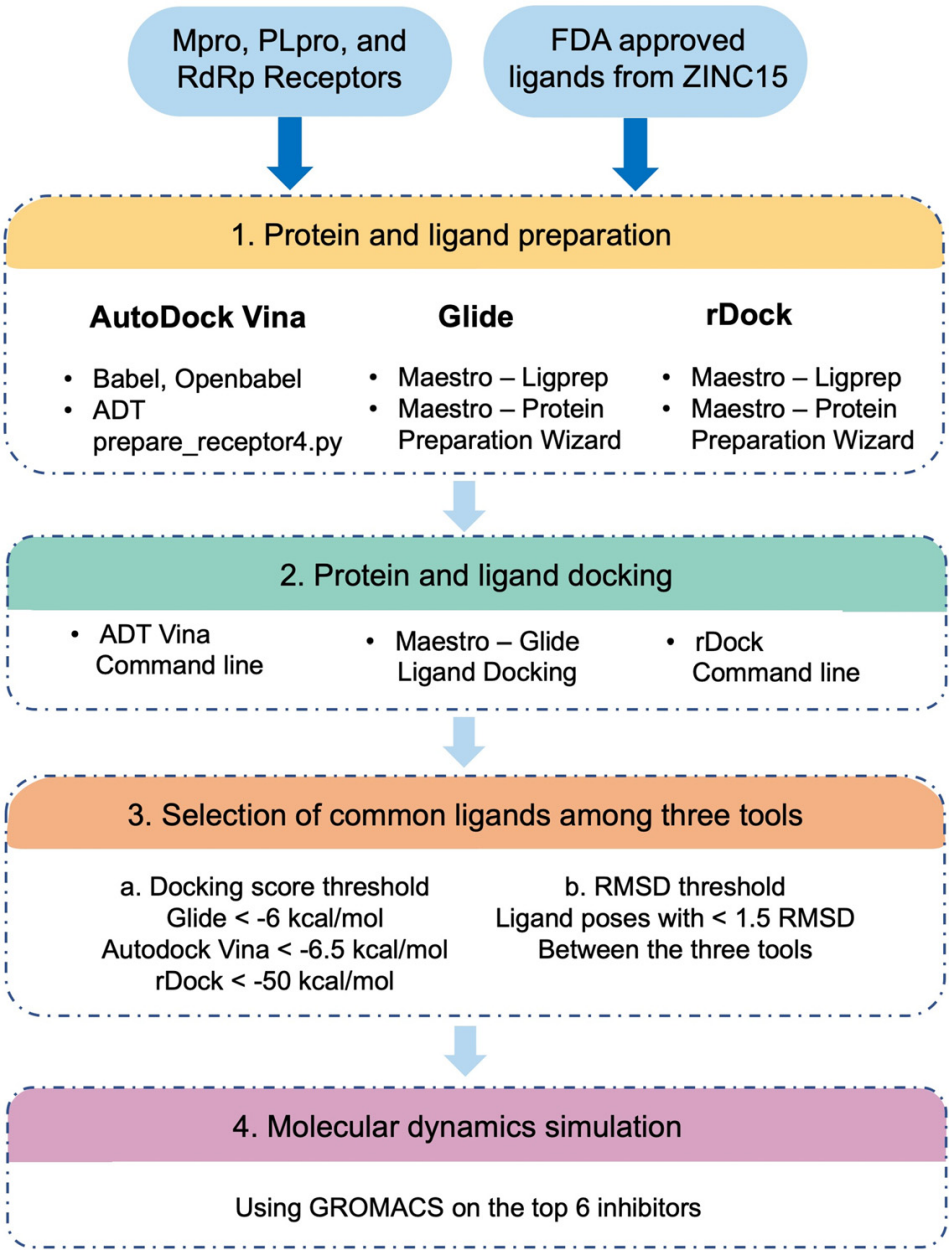
General scheme of the docking protocol.

To define the binding sites of the receptors, we retrieved co-crystallized ligand structures of known inhibitors for each of the receptors and used Glide's Receptor Grid Generation to generate grid files at the centroid for each ligand structure. The grid information was set as follows: Mpro N3 ligand, coordinates (−11.386, 12.409, 68.831) with box sizes of 32, 54,34 Å; PLpro TTT, coordinates (1.02, 21.89, 30.07) with box sizes of 22, 22, 22 Å; and RdRp RMP, coordinates (91.68, 92.49, 103.85) with box sizes of 17, 17, 17 Å. We re-docked the known inhibitors for each protein with our grid box information to confirm the chosen grid box information. The docking tools were set to generate 10 poses for each of the ligands to be docked to the protein binding site. Once the docking was completed, the ligand poses with lower than −6.5, −6, and −50 kcal/mol docking scores from AutoDock Vina, Glide, and rDock, respectively, were kept. Then, the ligand poses that passed the docking score threshold from the three docking tools were selected. The RMSD values between the same docked poses of the same ligand in the selected list were calculated and those poses with a RMSD value lower than 1.5 among the ADT Vina, Glide, and rDock were considered as potential inhibitors (Fig. 1). The potential inhibitors were sorted based on the Glide docking scores. The docking processes were done using written in-house scripts. All visualizations were done using Schrodinger Maestro^37^ and PyMOL (https://pymol.org/).

### Molecular dynamics simulation

Molecular dynamics (MD) simulations for six ligands from virtual screening analyses on Mpro, PLpro, and RdRp were carried out using GROMACS v5.1.4 (http://gromacs.org)^40,41^ for a period of 50 ns of timescale to investigate the stability of the docked ligand-protein complexes. The complexes were solvated in a cubical box, where the minimal distance between any ligand atom and the edge of the box was 10 Å. These ligand-protein complexes were prepared using GROMOS96 53a6 force field,^42^ and Na^+^/Cl^−^ ions added to neutralize the system and balance the charges. The initial energy minimization of the system was conducted using 5000 steps of the steepest descent algorithm via force convergence with less than 1000 kcal/mol/nm. Once the initial minimization was completed, the entire system was equilibrated for 5 ns at 300 K degree and 1 bar pressure using canonical (NVT) and the isothermal-isobaric (NPT) ensembles.^43,44^ The thermostat coupling was set with a reference temperature of 300 K degree using a Berendsen thermostat, and pressure coupling was set at 1.0 bar reference pressure using Parrinello-Rahman along with periodic boundary conditions with cut-offs for Lennard-Jones and Coulomb interactions. The Particle-Mesh Ewald (PME) algorithm was used for dialing with long-range interactions, and the final MD simulation was performed at 50 ns timescale for six ligand-protein complexes. The time step used for the simulation was 2 fs and coordinates were stored at every 10 fs. This was how the root-mean-square deviation (RMSD) and root-mean-square fluctuation (RMSF) were generated.

## Results

### Binding of 1615 FDA-approved drugs to SARS-CoV-2 Mpro, PLpro, and RdRp

We downloaded 1615 available FDA-approved drugs from the ZINC15 database,^36^ with a high-quality model of PLpro^35^ (based on 3E9S) and crystal structures of Mpro (6LU7) as well as RdRp (7BV2) with their co-crystalized inhibitors. Our analysis started in February 2020, and at the time 6LU7 was the only available SARS-CoV-2 Mpro structure with a co-crystalized inhibitor. However, by the end of our analysis, several other structures of Mpro became available on the Protein Data Bank (PDB) websites. We included 10 other Mpro structures with co-crystalized inhibitors: 7BGY, 6W63, 6XBI, 6XBH, 6XBG, 6WTT, 7BUY, 6M0K, 6LZE, and 6XFN to ensure that docking analysis with only one specific Mpro structure (6LU7 in our case) would not affect the results of the Mpro docking analysis. The 10 other Mpro structures were superimposed on 6LU7, and we found that the topologies of the structures were very similar, and the binding pocket residues were highly conserved between 6LU7 and the Mpro structures (Supplementary Fig. 1). The superimposition of the binding pocket residues of four Mpro structures (7BGY, 6W63, 6WTT, and 6LU7) in tube and stick styles with N3 inhibitor from 6LU7 in the pocket side are illustrated in Supplementary Fig. 1b. Thus, we continued our analysis based on the 6LU7 structure for Mpro.

**Figure 2.**
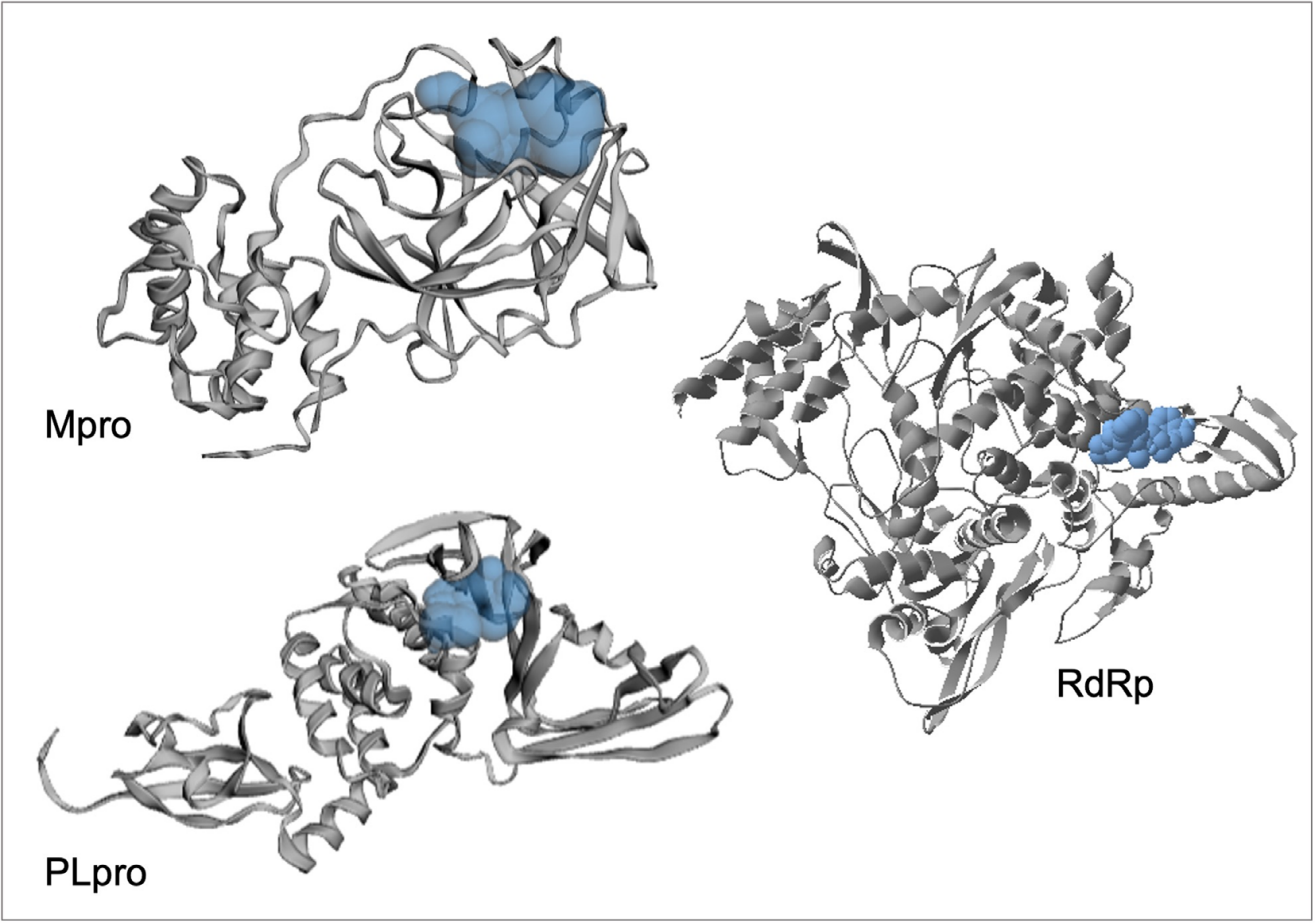
Cartoon representation of the SARS-CoV Mpro, PLpro, and RdRp protein structures with their pocket binding sites. Receptors are represented by gray ribbons. Pocket sites are shown as light blue bobbles.

^2+^ ^2+^ ^2+^Before starting docking analysis of the FDA-approved drugs, we redocked the co-crystalized ligand on its receptor to validate the docking parameters. Supplementary Fig. 2 illustrates the re-docking of each co-crystalized ligand N3, TTT, and remdesivir monophosphate (RMP) on their proteins, Mpro (top), PLpro (middle), and RdRp (bottom), respectively. The co-crystalized ligands are represented in yellow. We used AD Vina (left with the docked ligand in purple), Glide (middle with the docked ligand in green), and rDock (right with the docked ligand in cyan). The RMSD values for both Mpro and RdRp were lower than 2, and RMSD values for RdRp were less than 3, which were in the acceptable range. ^48,49^ The slightly higher values for RdRp are likely because of Mg ions present in the RdRp 7BV2 structure.^48^ As for the phosphate group of the co-crystalized ligand in 7BV2, remdesivir monophosphate (RMP) interacts with Mg ion, and it is believed that the inhibition of RdRp by remdesivir was most likely a result of interaction between the RdRp side chains Lys545 and Arg555 with remdesivir. After the removal of Mg from the structure for docking purposes, Arg555 formed an extra H-bond with the phosphate group of RMP, which led to a slight change in the directionality of the ligand.^50^Supplementary Fig. 3 illustrates the interactions between Mpro, PLpro, and RdRp with their co-crystalized ligands, respectively, in more detail.

We used three docking and virtual screening tools to obtain the accuracy of docking analyses: AutoDock Vina (AD Vina),^45^ Glide,^46^ and rDock.^47^ We used these tools to predict the interactions of ligands with each of the three proteins. Fig. 2 illustrates cartoon structures of Mpro, PLpro, and RdRp with their binding pockets colored in blue, which we used for our docking analysis. Fig. 1 illustrates the workflow used in this study.

### Potential SARS-CoV-2 main protein (Mpro) inhibitors

**Figure 3.**
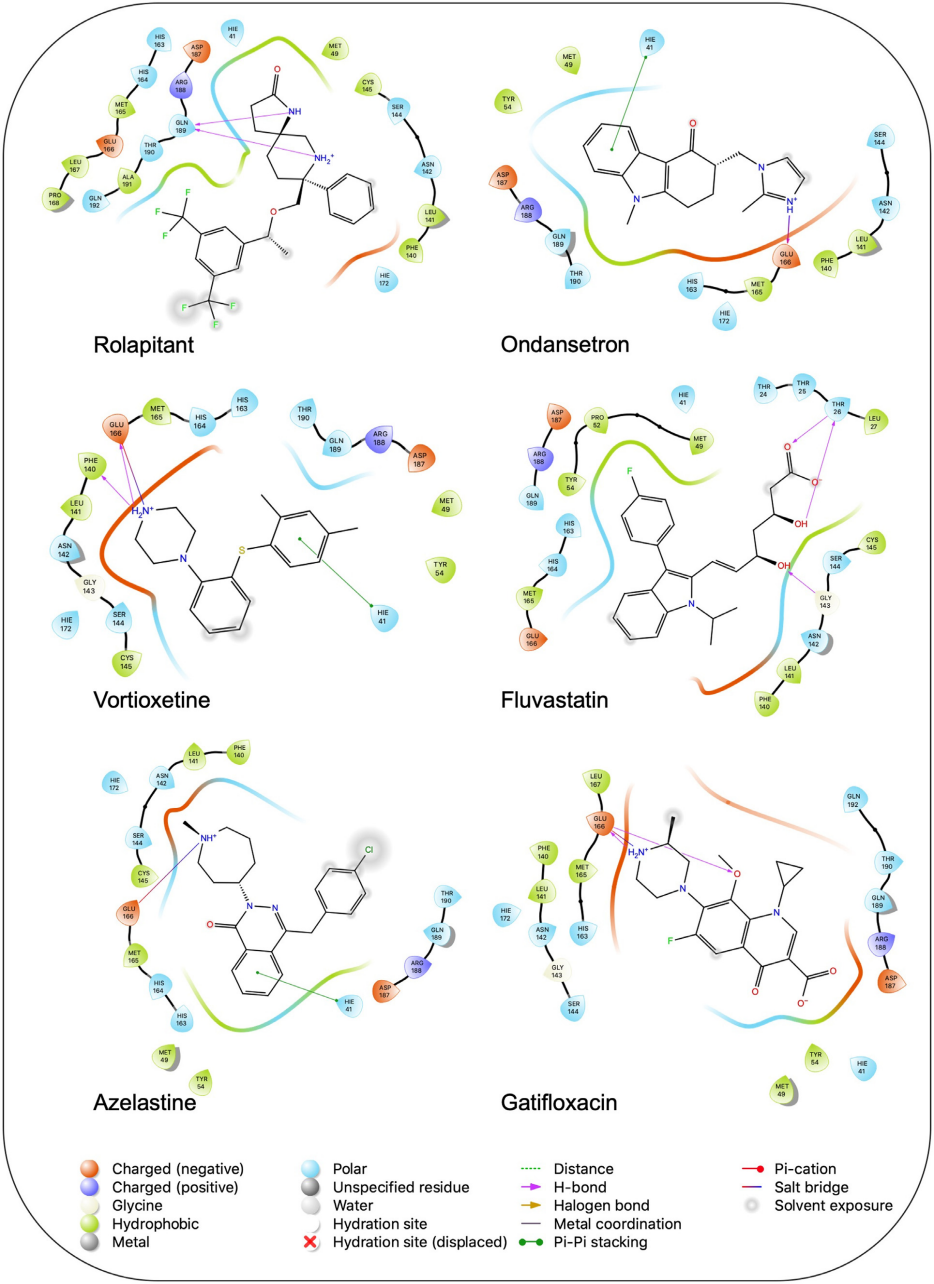
Interaction of the potential drugs with the COVID-19 Mpro protein. Ligands are shown as sticks. H-bonds between the receptor and ligands are shown as pink lines.

Based on our docking analysis, the potential ligand candidates that may inhibit SARS-CoV-2 Mpro are listed in Table 1, which shows the Glide docking scores. The list consists of antiemetics, antifungals, antiepileptics, antibiotics, an antidepressant, and an antihistamine, which all have minor side effects. The docking score values of the top 10 hits against Mpro were in the range −7.8 to −6.839, −8.4 to −6.9, and −84.90 to −51.37 kcal/mol for Glide, AD Vina, and rDock, respectively. The interactions of protein-ligand structures with low docking scores are illustrated in Fig. 3. All structures in our list of Mpro inhibitors (Table 1) formed H-bond interactions with Thr26, Phe140, Gly143, Glu166, and Gln189, and π-stacking interaction with His140, similar to that of N3 in Mpro.

**Table 1.** List of ligands that have low binding energy with the COVID-19 Mpro protein.

	Zinc ID/Drug name	2D structure	Pharmacological	Docking score (kcal/mol)		
				Glide	AutoDock Vina	rDock
1	ZINC000003816514/Rolapitant	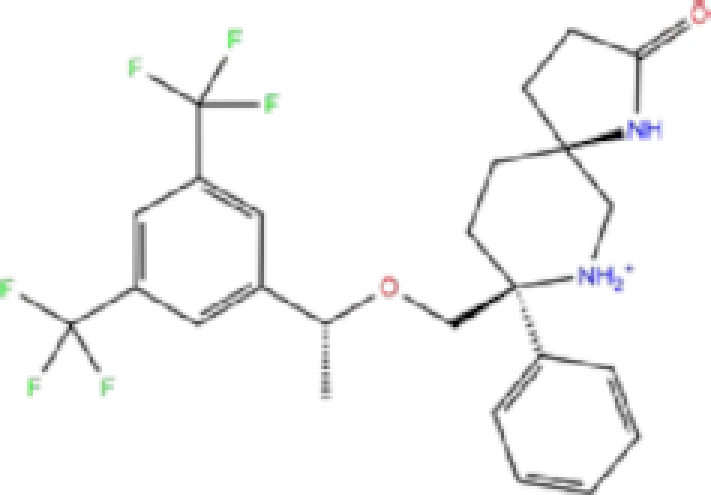	Antiemetic	−7.83	−7.2	−84.90
2	ZINC000000075126/Ondansetron	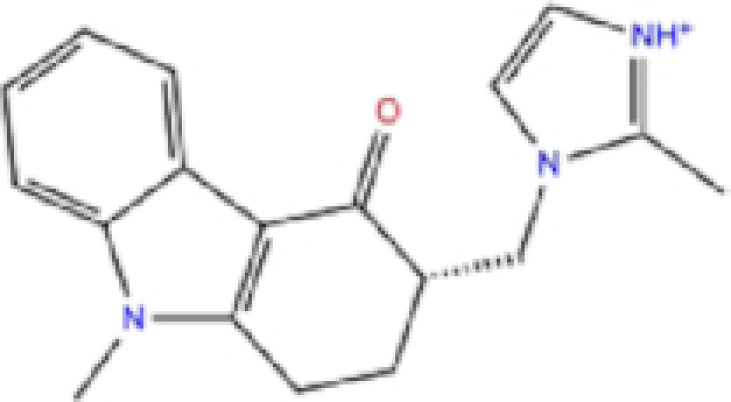	Antiemetic	−7.182	−6.9	−52.53
3	ZINC000034051848/Vortioxetine	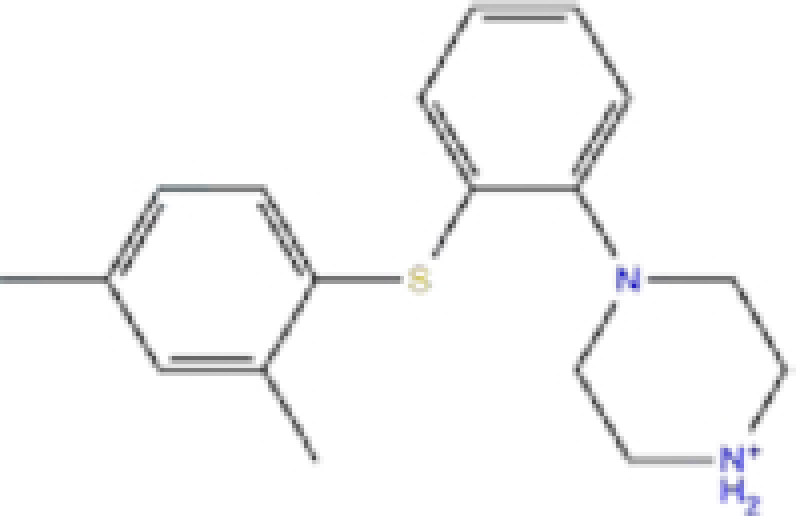	Antidepressant	−7.13	−6.9	−83.87
4	ZINC000001530639/Fluvastatin	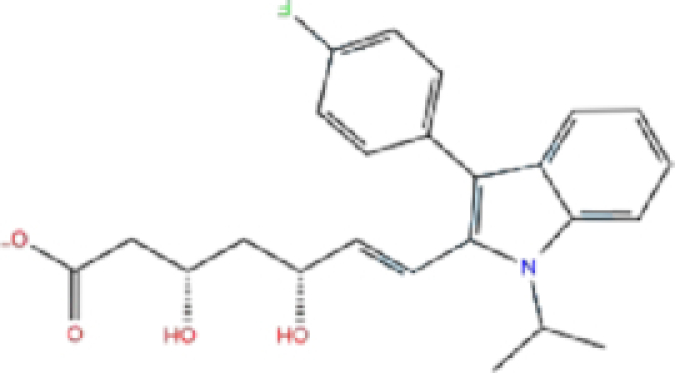	Antifungal	−7.096	−7.1	−84.21
5	ZINC000000897240/Azelastine	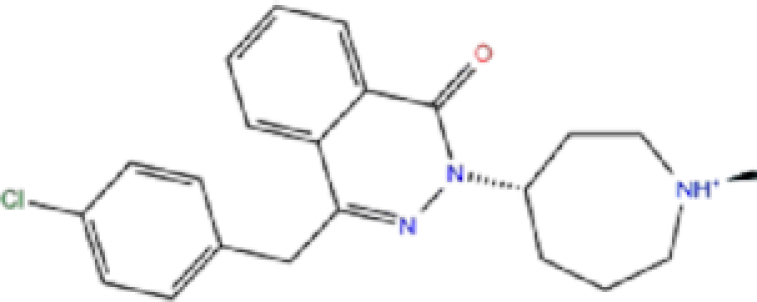	Antihistamine	−6.989	−8.3	−62.13
6	ZINC000038197764/Gatifloxacin	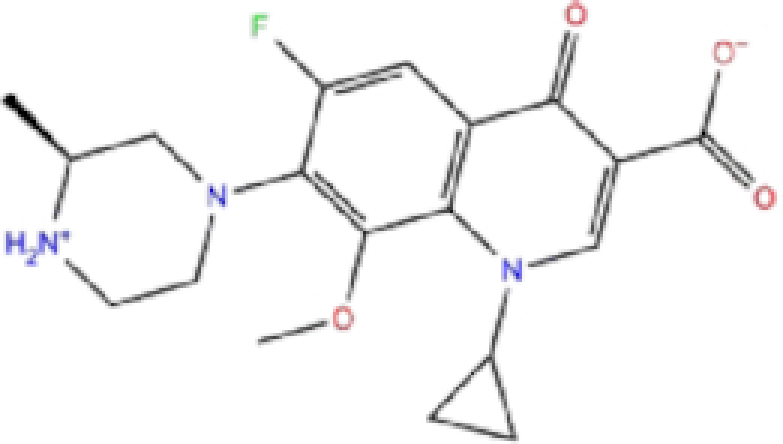	Antibiotics	−6.984	−7.2	−73.24
7	ZINC000030691797/Perampanel	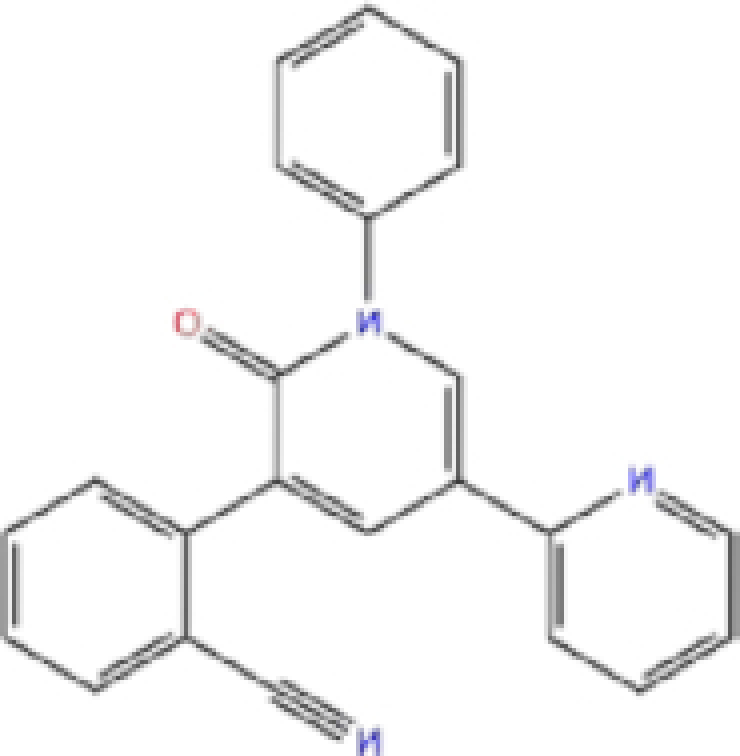	Antiepileptic	−6.965	−8.4	−51.37
8	ZINC000003812988/Butorfanol	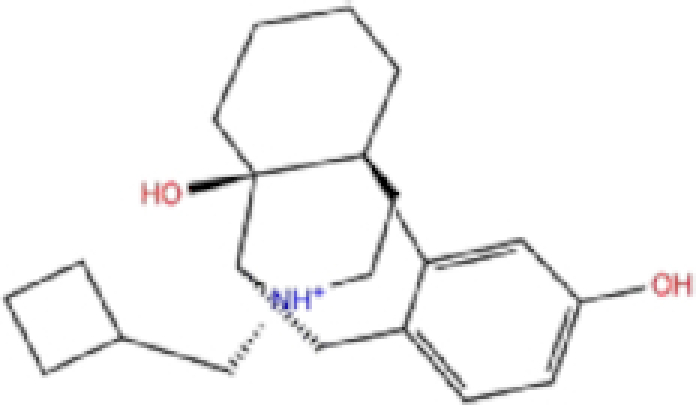	Antiepileptic	−6.956	−7.6	−60.21
9	ZINC000001530973/Butoconazole	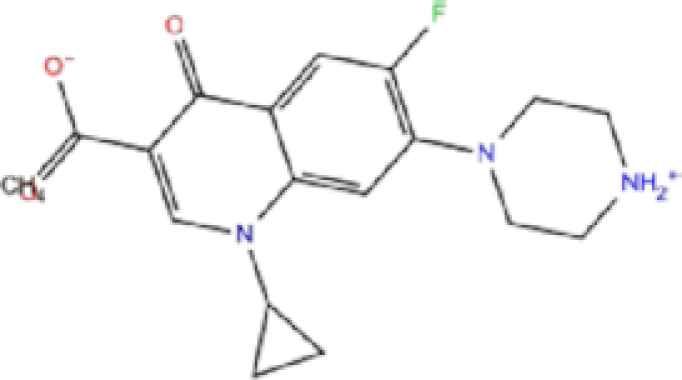	Antifungal	−6.868	−7	−76.23
10	ZINC000000020220/Ciprofloxacin	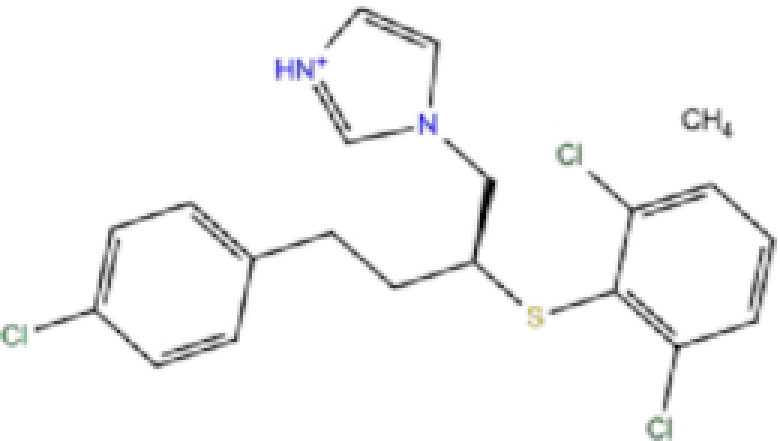	Antibiotic	−6.839	−7.7	−52.36

The results are sorted based on Glide docking score.

Rolapitant, a ligand in the top list towards Mpro, bound to Mpro with a glide docking score of −7.83 kcal/mol. It formed two hydrogen bonds with Mpro residues of Gln189 and three hydrophobic interactions with His41, Met165, and GLU166A, making it a promising Mpro inhibitor candidate. Ondansetron, vortioxetine, and azelastine were three ligands that formed π-stacking interaction with His140, which lowered their binding energy with Mpro. Fluvastatin is an antifungal, which formed two H-bonds with Thr26, and one H-bond with Gly143, and was recently identified as a SARS-CoV-2 Mpro inhibitor with low binding energy.^51^

### Potential SARS-CoV-2 papain-like protein (PLpro) inhibitors

**Figure 4.**
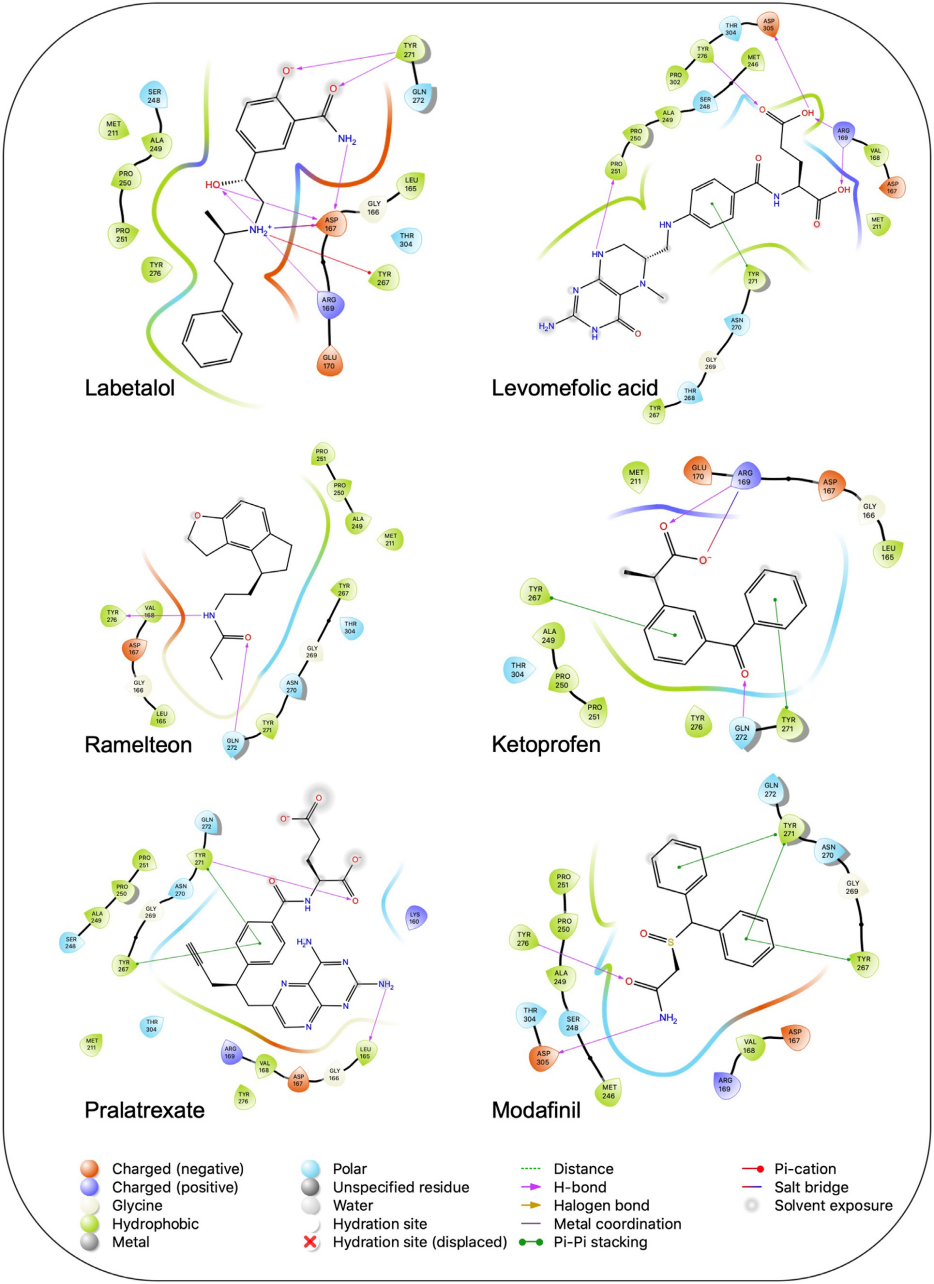
Interaction of the potential drugs with the COVID-19 PLpro protein. Ligands are shown as sticks. H-bonds between the receptor and ligands are shown as pink lines.

The potential inhibitors of SARS-CoV-2 PLpro, based on our docking analysis, are listed in Table 2. The docking scores of the top candidates of PLpro structures were −6.0 to −7.01, −6.7 to −8.4, and −51.98 to −78.51 kcal/mol for Glide, AutoDock Vina, and rDock, respectively. Figure 4 shows the interactions of the potential inhibitors with PLpro binding residues. In summary, most of the ligands in the top list of PLpro formed H-bonds with Arg169, Tyr271, Gln272, and Tyr276, and the majority of the structures formed π-stacking interactions with Tyr271, Tyr267.

**Table 2.** List of ligands that have low binding energy with the COVID-19 PLpro protein.

	Zinc ID/Drug name	2D structure	Pharmacological	Docking score (kcal/mol)		
				Glide	AutoDock Vina	rDock
1	ZINC000000004319/Labetalol	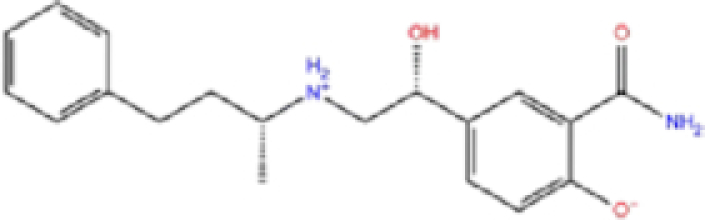	Hypertension treatment	−7.01	−7.4	−65.89
2	ZINC000002005305/Levomefolic acid	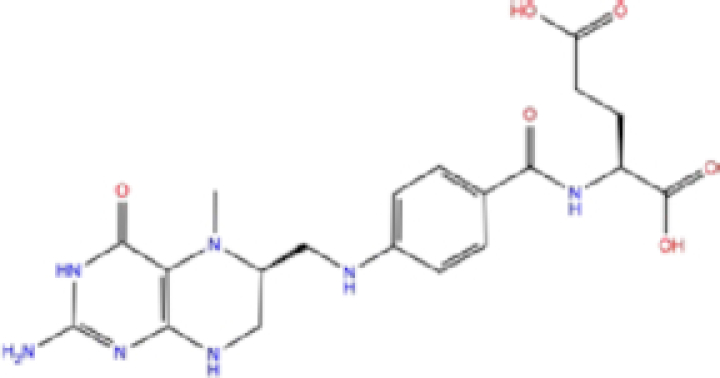	Active form of folic acid	−6.665	−7.3	−66.43
3	ZINC000003960338/Ramelteon	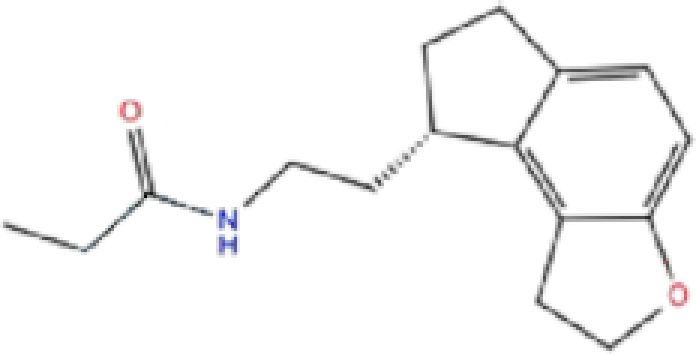	Sedative	−6.568	−7.7	−74.70
4	ZINC000000002272/Ketoprofen	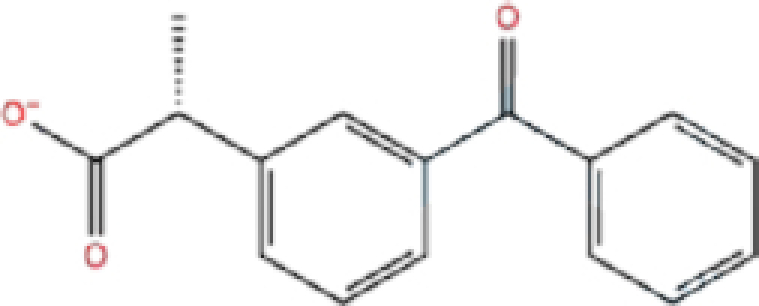	Anti-inflammatory	−6.311	−7.5	−63.42
5	ZINC000011616925/Pralatrexate	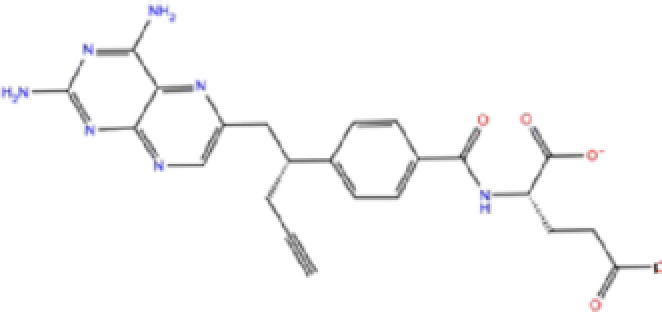	Chemotherapy	−6.3	−8	−75.51
6	ZINC000000006156/Modafinil	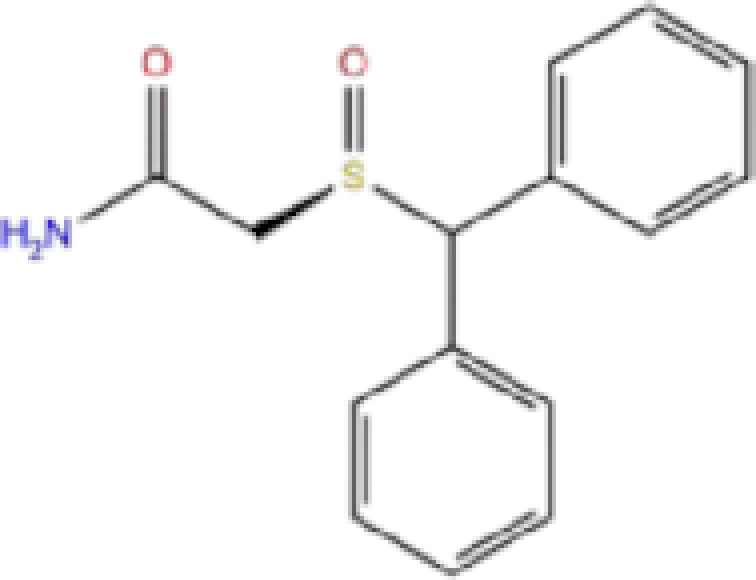	Stimulant	−6.245	−6.9	−66.94
7	ZINC000004392649/Tasimelteon	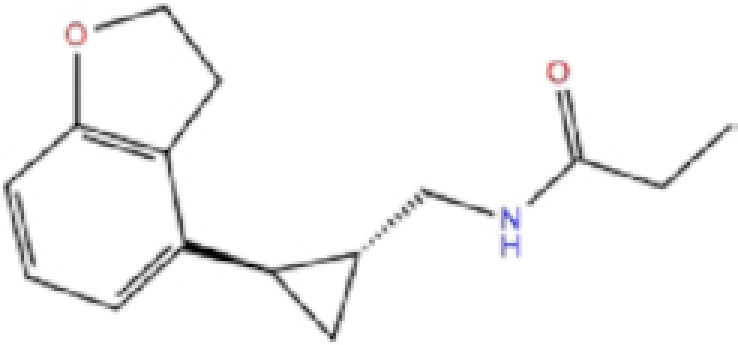	Wake disorder treatment	−6.145	−6.9	−59.32
8	ZINC000004228257/Tetrahydrobiopterin	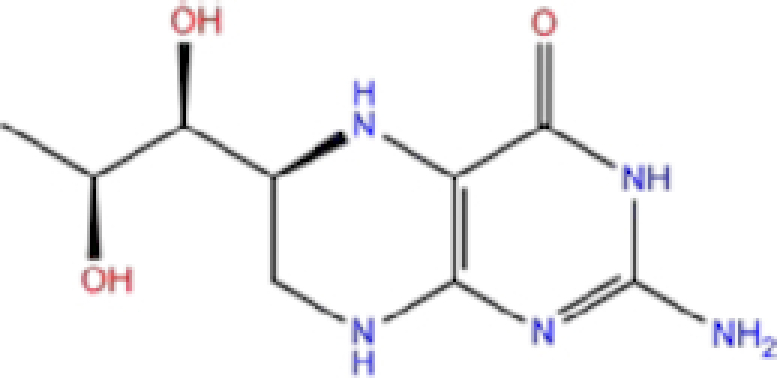	Dietary supplement	−6.042	−6.7	−51.98
9	ZINC000002570817/Bromfenac	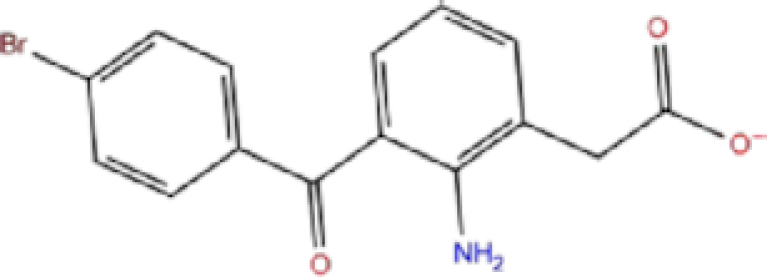	Anti-inflammatory	−6	−7.7	−78.51
10	ZINC000040430143/Olaparib	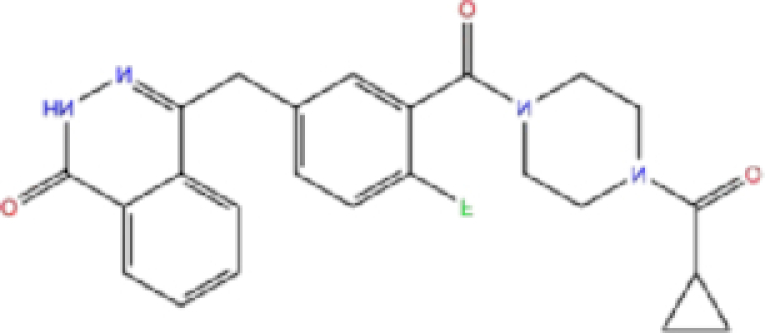	PARP inhibitor	−6.1	−8.4	−60.34

The results are sorted based on Glide docking score.

Labetalol formed two H-bonds with each of the three residues, i.e. Asp167, Arg169, and Tyr271, with a total of six H-bonds. It also formed a pi-cation interaction with Tyr267 along with six hydrophobic interactions with residues Leu165, Asp167, Pro251, Tyr267, Gln272, and Thr304, resulting in a ligand-receptor complex with low binding energy. Levomefolic acid is a ligand with lower binding energy, and it formed three H-bonds with Arg169, Asp305, and Tyr276, along with a π-stacking interaction with Tyr271. Levomefolic acid, ketoprofen, pralatrexate, and modafinil formed up to three π-stacking interactions with Tyr271 and Tyr267. Labetalol, levomefolic acid, ramelteon, modafinil, tetrahydrobiopterin, and bromfenac are drugs with minor side effects, and these drugs should be further studied as potential SARS-CoV-2 inhibitors.

### Potential SARS-CoV-2 RNA-dependent RNA polymerase (RdRp) inhibitors

The potential inhibitors of RdRp are listed in Table 3, and all of these drugs have minor side effects except folinic acid. The docking scores ranged from −6.016 to −7.17, −6.5 to −8.3, and −52.37 to −8.2 for Glide, AutoDock Vina, and rDock, respectively.

**Table 3.** List of ligands that have low binding energy with the COVID-19 RdRp protein.

	Zinc ID/Drug name	2D structure	Pharmacological	Docking score (kcal/mol)		
				Glide	AutoDock Vina	rDock
1	ZINC000009212427/Leucal (Leucovorin)	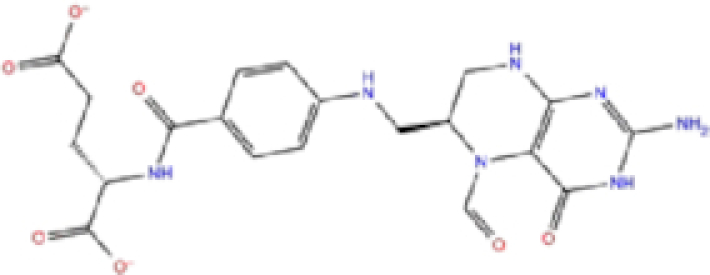	Detoxification	−7.17	−8.2	−74.27
2	ZINC000008220909/Natamycin	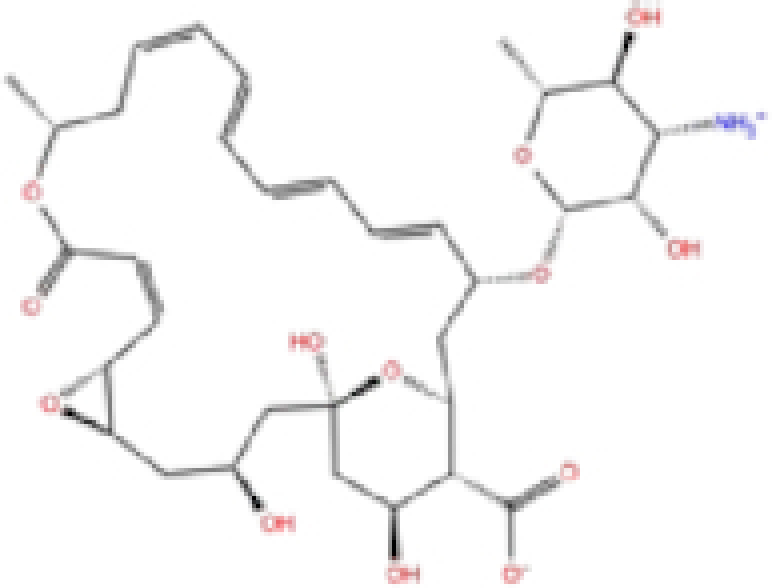	Antifungal	−7.126	−7.8	−64.73
3	ZINC000150338698/Capastat	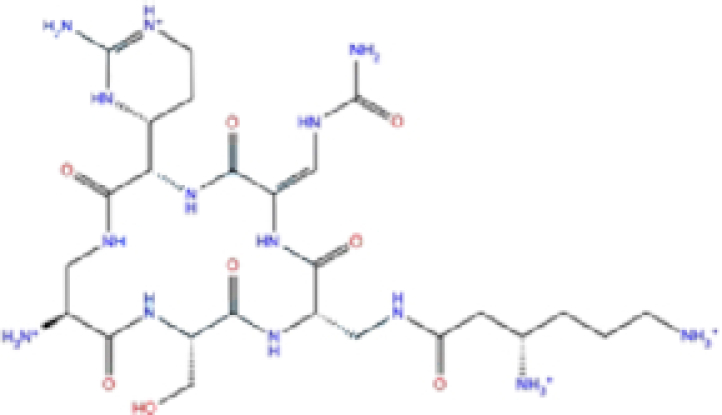	Antibiotic	−6.604	−6.5	−59.60
4	ZINC000029571072/Isavuconazonium	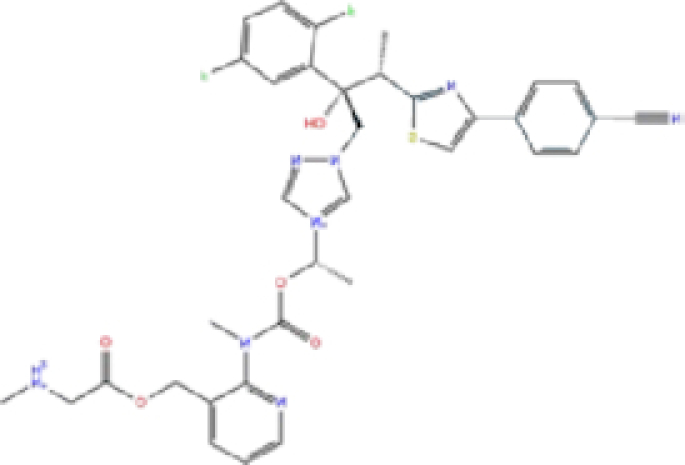	Antifungal	−6.524	−7.2	−53.96
5	ZINC000009212428/Folinic acid	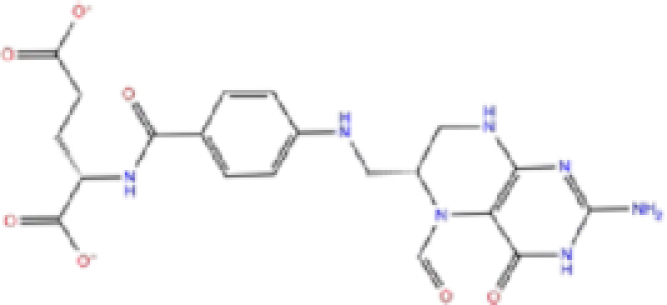	Chemotherapy	−6.299	−7.2	−70.09
6	ZINC000008577218/Folic acid	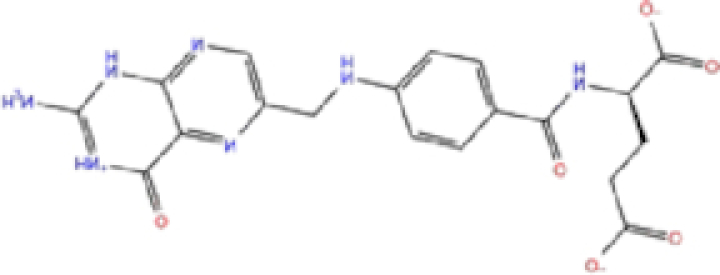	Vitamin	−6.016	−8.3	−52.37

The results are sorted based on Glide docking score.

**Figure 5.**
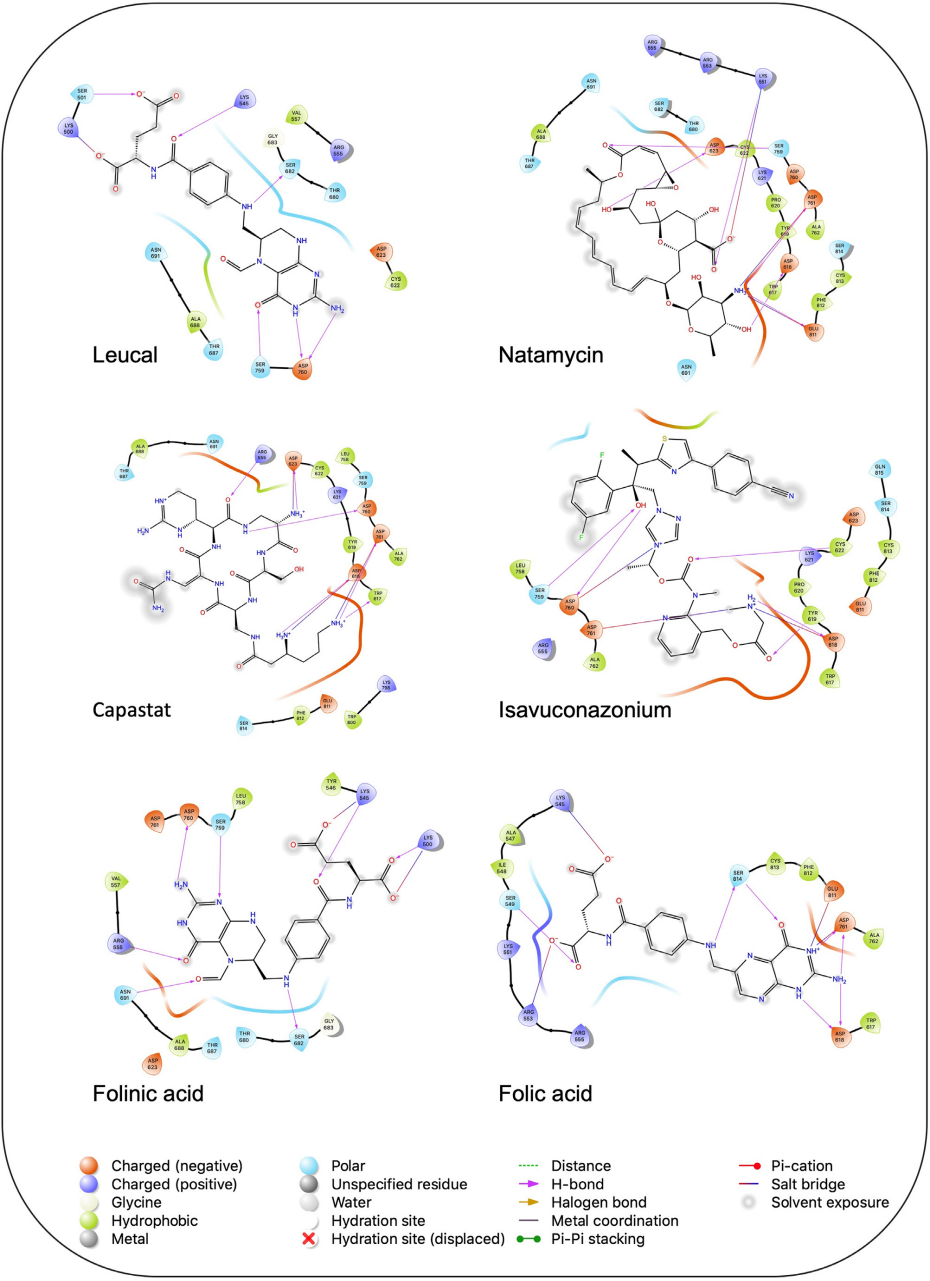
Interaction of the potential drugs with the COVID-19 RdRp protein. Ligands are shown as sticks. H-bonds between the receptor and ligands are shown as pink lines.

A schematic interaction of the protein-ligand complexes is presented in Fig. 5. In summary, all structures formed at least five H-bonds with RdRp pocket residues, and the majority of these interactions occurred at Lys545, Arg555, Asp618, Ser682, Ser759, Asp760, Asp761, and Glu811. Leucal, one of the top hits for RdRp, formed six H-bonds with Ser501, Lys545, Ser682, Ser759, and Asp760 (two bonds). Furthermore, it formed a salt bridge interaction with Lys500, lowering its docking score to −7.17 kcal/mol. A recent study suggested leucal as a potential SARS-CoV-2 Mpro inhibitor.^52^

### Comparison with promising COVID-19 drugs

**Figure 6.**
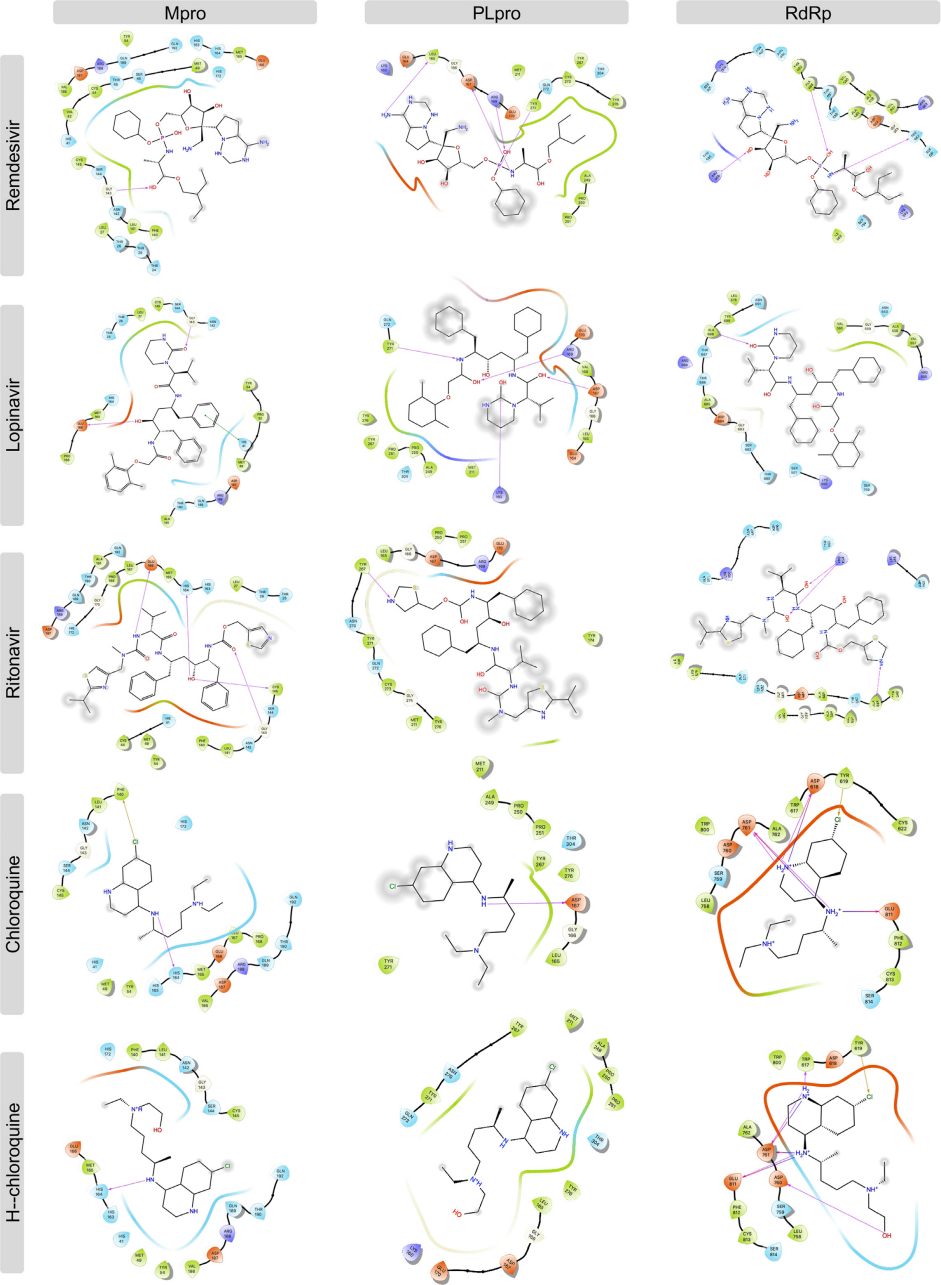
Interaction of the potential drugs with the COVID-19 Mpro, PLpro, and RdRp proteins. Ligands are shown as sticks. H-bonds between the receptor and ligands are shown as pink lines.

To compare our results with recently suggested SARS-CoV-2 drugs, we downloaded structures of remdesivir, lopinavir, ritonavir, chloroquine, and hydroxychloroquine; and we conducted docking analysis for these drugs against SARS-CoV-2 Mpro, PLpro, and RdRp proteins using three docking tools, AutoDock Vina, Glide, and rDock. Results of our docking analysis are listed in Table 4 and the corresponding ligand-protein interactions are presented in Fig. 6.

**Table 4.** Results of docking analysis on promising SARS-CoV-2 drugs.

	Binding energy (kcal/mol)								
Drugs	Mpro			PLpro			RdRp		
	Glide	AD Vina	rDock	Glide	AD Vina	rDock	Glide	AD Vina	rDock
**Remdesivir**	−7.40	−7.9	−74.32	−6.87	−6.1	−75.01	−7.59	−8.1	−82.22
**Lopinavir**	−7.26	−9.3	−81.57	−7.10	−8.6	−69.02	−7.61	−10.1	−74.17
**Ritonavir**	−6.83	−7.6	−78.29	−6.90	−8.4	−66.49	−6.73	−8.5	−80.01
**Chloroquine**	−6.00	−5.8	−66.32	−5.83	−6.0	−64.72	−5.91	−5.7	−56.53
**Hydroxy-chloroquine**	−5.98	−5.8	−67.01	−5.83	−6.1	−63.28	−5.90	5.7	−56.40

Remdesivir, a recently FDA-approved SARS-CoV-2 drug,^19^ has been shown to target both RdRp and Mpro with low binding energies.^19,20,53–56^ Interestingly, our analysis showed a lower binding energy for rolapitant/Mpro (−7.83 kcal/mol, Glide) compared to remdesivir/Mpro (−7.4 kcal/mol, Glide). Ondansetron, the second drug in our list of potential Mpro inhibitors, shows slightly higher binding energy (−7.18 kcal/mol, Glide) compared to that of remdesivir/Mpro. Furthermore, leucal/RdRp showed low AD Vina binding energy (−8.2 kcal/mol) compared to remdesivir/RdRp (−8.1 kcal/mol). Natamycin/RdRp came second in our list with AD Vina −7.8 and Glide −7.126 kcal/mol binding energies, which were slightly higher than remdesivir/RdRp. Remdesivir has not been suggested as an active inhibitor of PLpro and the binding energies with all three docking tools for remdesivir/PLpro were higher than for labetalol and levomefolic acid.

Lopinavir and ritonavir are suggested inhibitors of Mpro.^18,57^ Our analysis showed slightly lower binding energy for these drugs with RdRp (−10.1 and −8.5 kcal/mol, AD Vina) compared to Mpro (−9.3 and −7.6 kcal/mol, AD Vina), suggesting that they could be potential inhibitors of RdRp as well. Chloroquine and hydroxychloroquine have been suggested as potential inhibitors of PLpro,^27^ but our docking analysis showed a high binding energy for these ligands with all three SARS-CoV-2 proteins, suggesting either there was no inhibitory activity against SARS-CoV-2 or the antiviral effects of chloroquine might be mainly at the entry-level rather than the post-entry stage.

### Ensemble docking of Mpro structures

During the course of our analysis, several complexes of SARS-CoV-2 Mpro became available. To confirm the accuracy of our docking results based on one specific Mpro structure (6LU7), we performed ensemble docking for 10 Mpro structures with co-crystalized ligand inhibitors (7BGY, 6W63, 6XBI, 6XBH, 6XBG, 6WTT, 7BUY, 6M0K, 6LZE, and 6XFN), and 10 potential Mpro inhibitors that were identified by our virtual screening analysis using Schrödinger Virtual Screening Workflow (Table 1). As shown in Supplementary Fig. 1c, all 10 ligand structures had low glide docking scores ranging from −6.461 to −7.843 kcal/mol.

### Molecular dynamics (MD) simulation

**Figure 7.**
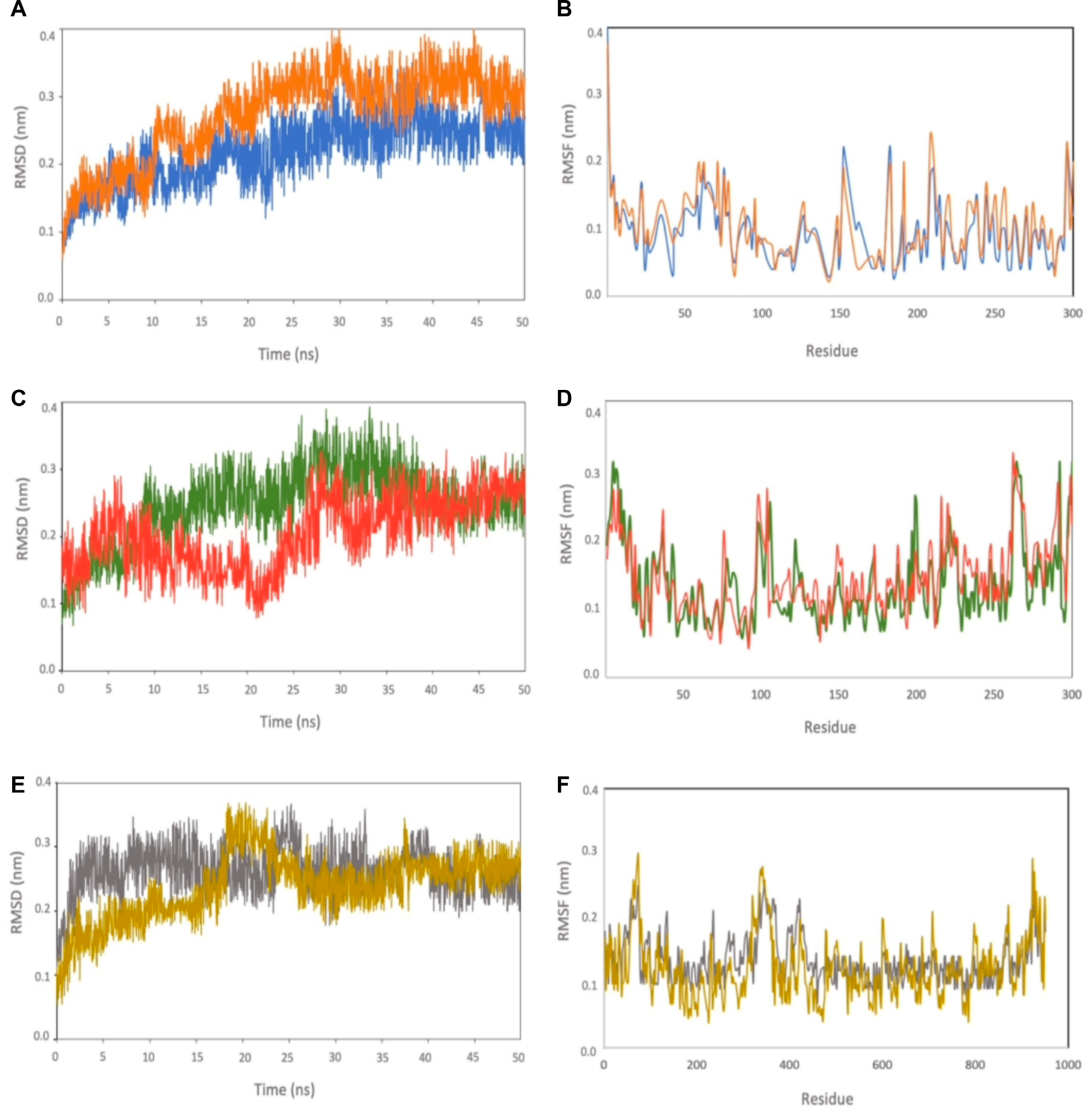
RMSD values derived from molecular dynamics simulation. Root-mean-square deviations (RMSD) of the backbone and root-mean-square fluctuations (RMSFs) of Cα over 50 ns of MD simulation for (A, B) Mpro-rolapitant (blue) and Mpro-ondansetron (orange), (C, D) PLpro-labetalol (red) and PLpro-levomefolic (green) (E, F) RdRp-leucal (gray) and RdRp-natamycin (gold).

To validate and confirm the stability of the suggested protein-ligand complexes, we performed MD simulation at 50 ns for the top six SARS-CoV-2 inhibitor-protein complexes identified from our virtual screening studies. We selected the top two potential ligands from the list of potential inhibitors for Mpro, PLpro, and RdRp (Tables 1–3) including: rolapitant and ondansetron (Mpro inhibitors), labetalol and levomefolic acid (PLpro inhibitors), and leucal and natamycin (RdRp inhibitors). We computed the root-mean-square deviation (RMSD) and root-mean-square fluctuation (RMSF) for each complex (Fig. 7A, C, E). The RMSD value can predict the ligand-complex stability of the MD runs. A lower RMSD value indicates a higher protein complex stability. We calculated the RMSD of the complexes with respect to the Cα atom against the MD simulation time. Overall, the average RMSD for all six complexes were low, ranging from 2.12 to 2.83 Å for PLpro-labetalol and Mpro-ondansetron, respectively.

Mpro-rolapitant and -ondansetron complexes had average RMSD values around 2.18 and 2.83 Å, respectively (Fig. 7A). Mpro-rolapitant showed some fluctuations at around 17 and 45 ns. Mpro-ondansetron displayed fluctuations at 12 ns and 30 ns, and remained stable during the rest of the simulation period. The complexes of PLpro had average RMSD values around 2.12 and 2.46 Å for PLpro-labetalol and PLpro-levomefolic, respectively (Fig. 7C). The average RMSD values for RdRp-leucal and -natamycin complexes were 2.63 and 2.48 Å (Fig. 7E), slightly higher compared with those of PLpro and Mpro. Interestingly, the RMSD curves for RdRp complexes showed very similar patterns of conformation changes after 50 ns. Overall, the low average RMSD values observed in the six protein-ligand complexes indicated strong binding between the ligands and the proteins. To check the flexibility of the residues during molecular dynamics simulation, we calculated the RMSF of Cα-atoms for six complexes, and found that all systems displayed similar fluctuations (Fig. 7B, D, F). Overall, the RMSF values for all complexes were below 2 Å. Furthermore, the fluctuation curve for each protein-complex group was similar (Fig. 7B, D, F).

## Discussion

The pandemic of COVID-19 has become a global emergency. Scientists and physicians are searching for potential drugs for the treatment, and there is an urgent need to identify effective drugs with lower side effects to fight against SARS-CoV-2. Molecular docking is a promising computational tool in drug discovery and identifying potential drug candidates.^58^ Thus, we conducted molecular docking and virtual screening of 1615 FDA-approved drugs targeting three important non-structural proteins of SARS-CoV-2, main protease (Mpro), papain-like protease (PLpro), and RNA-dependent RNA polymerase (RdRp) using AutoDock Vina, Glide, and rDock. We identified a list of ligands that not only had low binding energy (potential high inhibitory activity) based on all three docking tools, but also had low RMSD values between the ligand poses. Molecular docking simulations confirmed the stability of the potential drugs ranked at the top of our list. Our results suggested six new FDA-approved drugs with lower binding energy as potential inhibitors against SARS-CoV-2 targets. Our suggested candidate ligands are antiemetics rolapitant and ondansetron for Mpro, labetalol and levomefolic acid for PLpro, and leucal and antifungal natamycin for RdRp. These six ligands can be considered as potential candidate drugs subject to further clinical studies.

Other in silico studies have also reported rolapitant and ondansetron as potential COVID-19 Mpro inhibitors by conducting different computational methods such as Movable Type (MT) Free Energy,^59^ Consecutive Histogram Monte Carlo (CHMC) sampling, and CB-Dock (http://cao.labshare.cn/cb-dock/),^60^ which have confirmed the stability of the protein-ligand complex stability with MD simulations.^61–64^ Consistent with other molecular docking studies,^27,65,66^ both labetalol and levomefolic acid were identified as potential PLpro inhibitors in our study. Recent studies found that labetalol had high inhibitory activity on SARS-CoV-2 spike protein, likely by a mechanism of changing ACE2 structure which may affect the recognition and interaction of ACE2 with viral spike.^67–69^ Levomefolic acid has also been reported as a potential inhibitor for RdRp,^70^ nevertheless our results did not show a high inhibitory activity of levomefolic acid against RdRp. Leucal with a glide −7.17 and AD Vina −8.2 kcal/mol binding energy, was at the top of the list of our potential RdRp inhibitors. There have not been any studies on the inhibitory activity of leucal with RdRp. Two in silico studies on Mpro inhibitors have suggested leucal as a potential inhibitor against Mpro as well;^52,71^ however, both of these studies were based on only one docking tool. Natamycin, in our list of potential RdRp inhibitors, has shown good inhibitory activity not only to RdRp, but also with SARS-CoV-2 helicase and PLpro.^72,73^

We conducted molecular docking analysis on five drugs that have been suggested as promising SARS-CoV-2 inhibitors: remdesivir, chloroquine, hydroxychloroquine, lopinavir, and ritonavir. Remdesivir was the first and only approved drug that has been suggested to inhibit RdRp and Mpro.^19,20,53–56^ Chloroquine/hydroxychloroquine and lopinavir/ritonavir, however, have been removed from the COVID-19 treatment protocols because of possible risks and uncertainty regarding their benefits, but are still being studied in clinical trials.^17,18^ Our docking results showed low binding energy for rolapitant/Mpro (−7.83 kcal/mol, Glide) compared with remdesivir/Mpro (−7.4 kcal/mol, Glide) and leucal/RdRp (−8.2 kcal/mol, AD Vina) compared with remdesivir/RdRp (−8.1 kcal/mol, AD Vina). Ondansetron/Mpro and natamycin/RdRp showed slightly higher binding energies compared with remdesivir/Mpro and remdesivir/RdRP, respectively, but the molecular dynamics results confirmed the stability of the ligand-protein complex, thus they can still be considered as potential COVID-19 inhibitors. Both labetalol and levomefolic acid showed lower binding energies with PLpro compared with remdesivir/PLpro, suggesting they may have better inhibitory activity against PLpro. Lopinavir and ritonavir were suggested as Mpro inhibitors;^18,57^ however, our analysis revealed lower binding energies for lopinavir/RdRp and ritonavir/RdRp of −10.1 and −8.5 kcal/mol (AD Vina) compared with lopinavir/Mpro and ritonavir/Mpro of −9.3 and −7.6 kcal/mol (AD Vina), which suggests that lopinavir and ritonavir could be potential inhibitors of RdRp as well. Chloroquine and hydroxychloroquine, reported in one study as PLpro inhibitors,^27^ have high binding energy (low inhibitory effect) with all three SARS-CoV-2 proteins in our analysis, suggesting that either they have no inhibitory activity against SARS-CoV-2 or that the antiviral effects of chloroquine may be mainly at the entry-level rather than the post-entry stage.

Our molecular docking and virtual screening have identified some potential new ligands, e.g., rolapitant, leucal, and labetalol, as promising inhibitors against SARS-CoV-2. We plotted the interactions of our suggested potential drugs with the SARS-CoV-2 proteins to further help in choosing the optimized drugs. To cross-validate our molecular docking and screening findings, we performed MD simulations that confirmed the ligand-complex stability for the candidates we identified. We acknowledge that computational docking analysis has its limitations, and that further laboratory and clinical studies are needed to validate the inhibitory effects of these candidates against SARS-CoV-2 as potential drugs for COVID-19.

## Supplementary Material

pbab001_Supplemental_File

## Data Availability

**PDB files of homology (docking) models can be found at** https://doi.org/10.7910/DVN/HD7HON.
