# Supplementary material for: Computational molecular docking and virtual screening revealed promising SARS-CoV-2 drugs
Source: Precis Clin Med. 2021 Jan 18;4(1):1–16. doi: 10.1093/pcmedi/pbab001 (PMC7928605; doi:10.1093/pcmedi/pbab001)
Supplement: pbab001_Supplemental_File [file pbab001_supplemental_file.pdf]

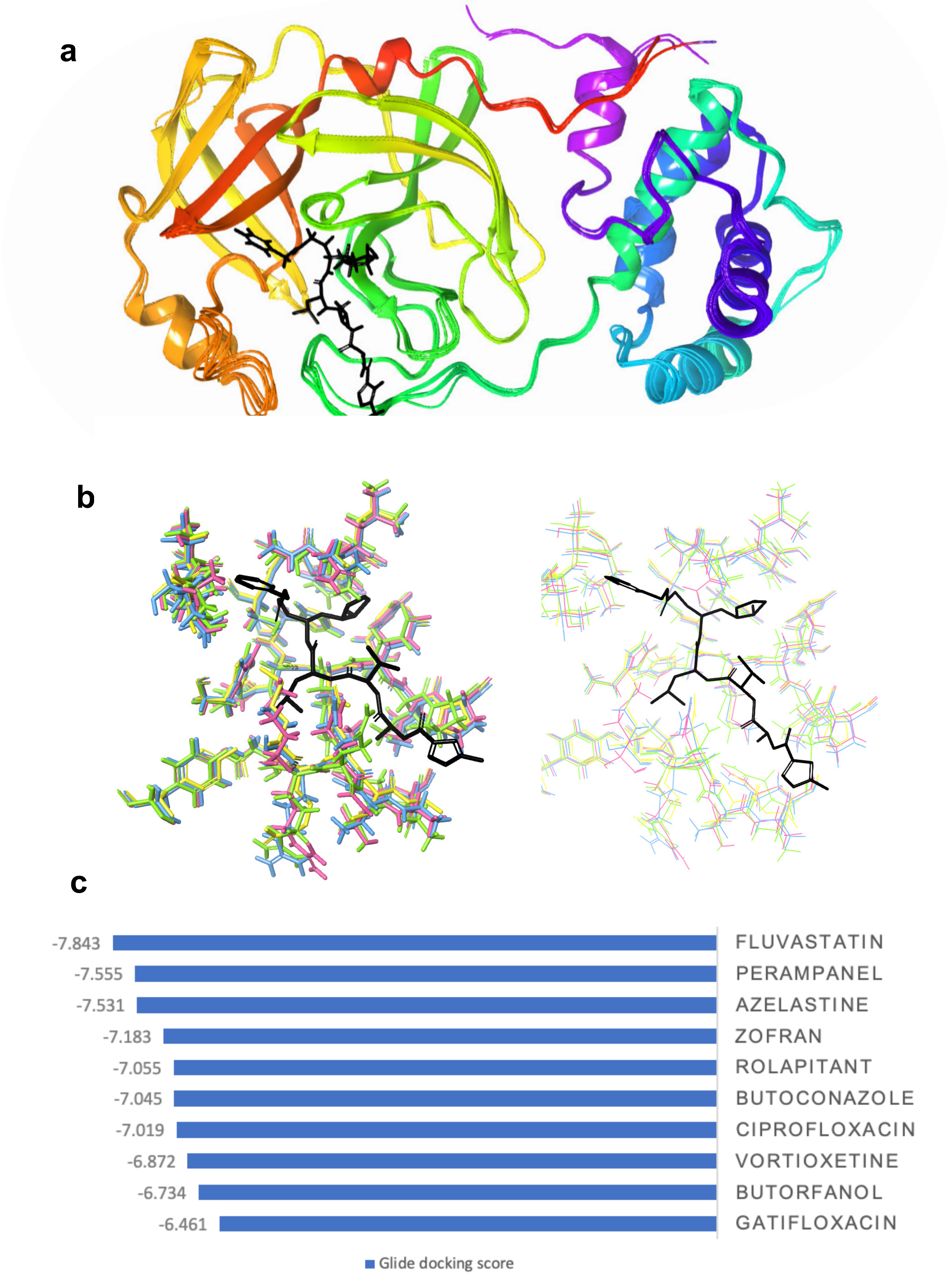

Supplementary Figure S1. **Structural similarity of SARS-CoV-2 Mpro proteases. (a)** Superimposition of ten SARS-CoV-2 Mpro structures (7BGY, 6W63, 6XBI, 6XBH, 6XBG, 6WTT, 7BUY, 6M0K, 6LZE, and 6XFN) represented in ribbons and N3 inhibitor from 6LU7 in black tube; **(b)** Superimposition of binding pocket residues of four Mpro structures (7BGY, 6W63, 6WTT, and 6LU7) in tube (left) and stick (right) styles with N3 inhibitor from 6LU7 in black tube; **(c)** Result of ensemble docking of top ten Mpro structures with top ten ligands with low binding energy with 6LU7 from our studies in **Table 2**. Results are sorted based on Glide docking score.

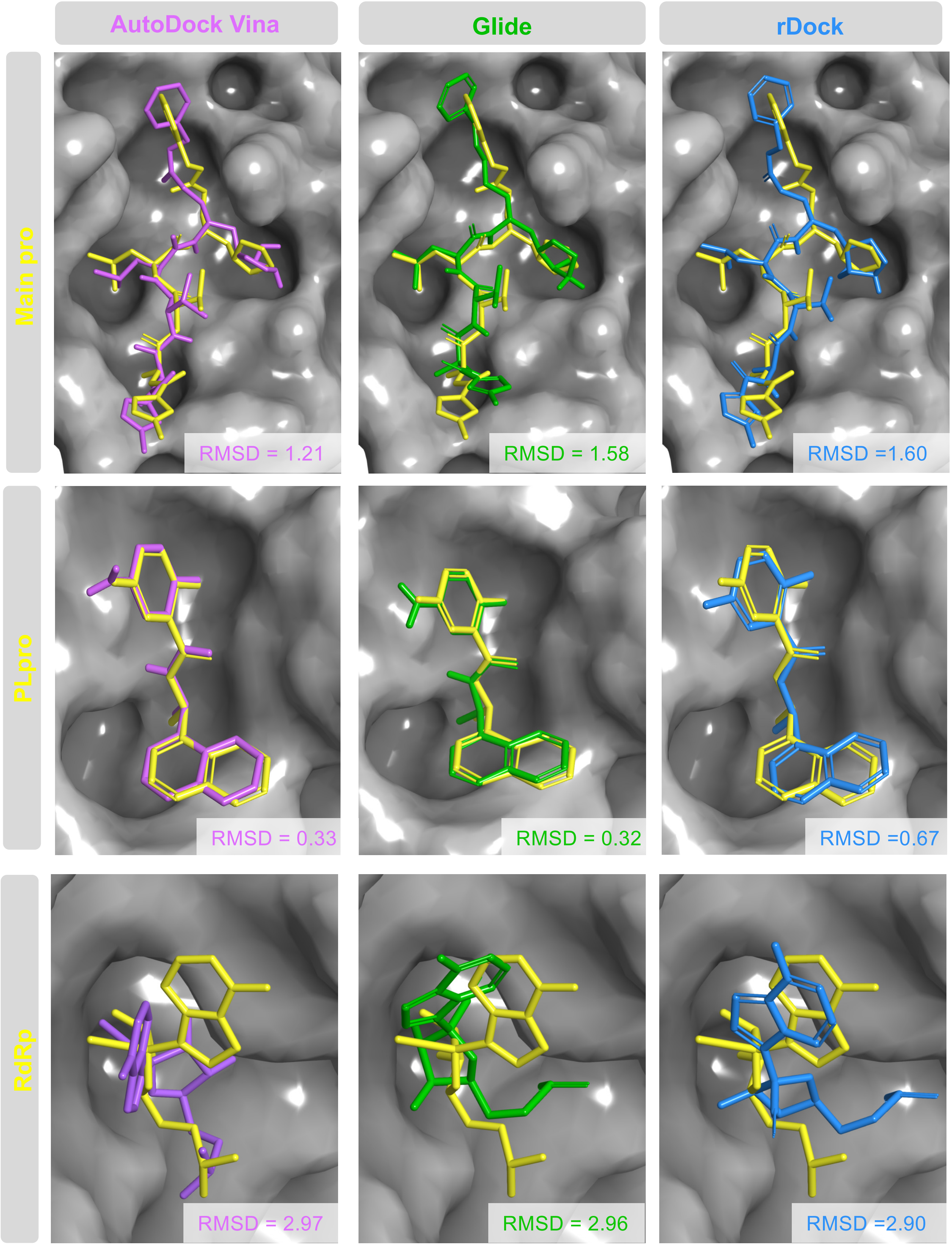

Supplementary Figure S2. Docking validation. Re-docking of co-crystalized ligands on Mpro (top), PLpro (middle), and RdRp (bottom) using three docking tools AutoDock Vina (left), Glide (middle), and rDock (right). Co-crystalized ligand is represented in yellow color and the docked forms are represented in purple, green, and blue for AutoDock Vina, Glide, and rDock, respectively.

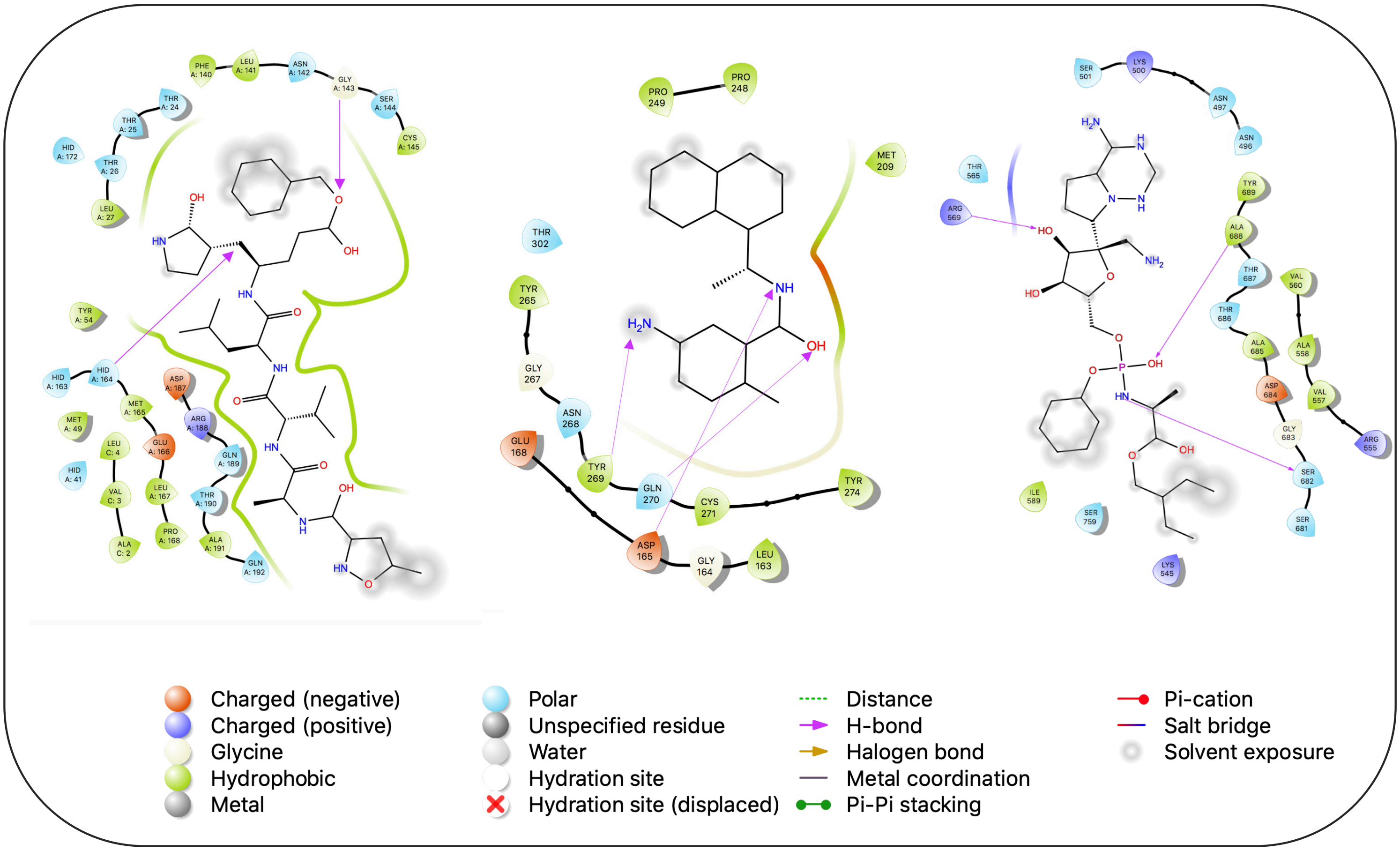

Supplementary Figure S3. Interaction of SARS-CoV-2 Mpro, PLpro, and RdRp proteins with their co-crystalized ligands. **(Left)** Mpro (6LU7) with N3; **(middle)** PLpro (3E9S) with TTT (5-amino-2-methyl-N-[(1R)-1-naphthalen-1-ylethyl] benzamide); **(right)** RdRp (7BV2) with Remdesivir.
